# Clinico-demographic characteristics and outcomes of radiation-induced sarcomas (RIS): a CanSaRCC study

**DOI:** 10.1177/17588359231198943

**Published:** 2023-09-28

**Authors:** Mauricio Fernando Ribeiro, Hagit Peretz Soroka, Zainab Bhura, Ian Hirsch, Jay Wunder, Peter Ferguson, Kim Tsoi, Savtaj Brar, Rebecca Gladdy, Carol Swallow, Peter Chung, Charles Catton, Philip Wong, Geoffrey Watson, Albiruni Ryan Abdul Razak, Abha A. Gupta, David Shultz

**Affiliations:** Division of Medical Oncology and Hematology, Princess Margaret Cancer Centre, University of Toronto, ON, Canada; Division of Medical Oncology and Hematology, Princess Margaret Cancer Centre, University of Toronto, ON, Canada; Division of Medical Oncology and Hematology, Princess Margaret Cancer Centre, University of Toronto, ON, Canada; Division of Medical Oncology and Hematology, Princess Margaret Cancer Centre, University of Toronto, ON, Canada; Department of Surgery, Mount Sinai Hospital, Sinai Health, Toronto, ON, Canada; Department of Surgical Oncology, Princess Margaret Cancer Centre, University of Toronto, ON, Canada; Department of Surgery, Mount Sinai Hospital, Sinai Health, Toronto, ON, Canada; Department of Surgical Oncology, Princess Margaret Cancer Centre, University of Toronto, ON, Canada; Department of Surgery, Mount Sinai Hospital, Sinai Health, Toronto, ON, Canada; Department of Surgical Oncology, Princess Margaret Cancer Centre, University of Toronto, ON, Canada; Department of Surgery, Mount Sinai Hospital, Sinai Health, Toronto, ON, Canada; Department of Surgery, Mount Sinai Hospital, Sinai Health, Toronto, ON, Canada; Department of Surgical Oncology, Princess Margaret Cancer Centre, University of Toronto, ON, Canada; Department of Surgery, Mount Sinai Hospital, Sinai Health, Toronto, ON, Canada; Department of Surgical Oncology, Princess Margaret Cancer Centre, University of Toronto, ON, Canada; Department of Radiation Oncology, Princess Margaret Cancer Centre, University of Toronto, ON, Canada; Department of Radiation Oncology, Princess Margaret Cancer Centre, University of Toronto, ON, Canada; Department of Radiation Oncology, Princess Margaret Cancer Centre, University of Toronto, ON, Canada; Division of Medical Oncology, Mount Sinai Hospital, Sinai Health, Toronto, ON, Canada; Division of Medical Oncology and Hematology, Princess Margaret Cancer Centre, University of Toronto, ON, Canada; Division of Medical Oncology, Mount Sinai Hospital, Sinai Health, Toronto, ON, Canada; Division of Medical Oncology and Hematology, Princess Margaret Cancer Centre, University of Toronto, ON, Canada; Department of Radiation Oncology, Princess Margaret Cancer Centre – University of Toronto, 610 University Avenue, Toronto, ON M5G 2M9, Canada

**Keywords:** angiosarcoma, breast sarcoma, radiation-induced cancers, radiation-induced sarcoma, radiotherapy, soft-tissue sarcoma

## Abstract

**Background:**

Radiation-induced sarcomas (RIS) tend to have aggressive behaviour and because of their rarity, the most appropriate management for these malignancies is uncertain.

**Objectives:**

Using the Canadian Sarcoma Research and Clinical Collaboration (CanSaRCC) database, a national sarcoma registry, we aimed to investigate prognostic factors and outcomes for RIS.

**Design:**

Retrospective study of RIS patients treated from 1996 to 2021 at three Canadian centres.

**Methods:**

RIS was defined as a sarcoma arising in a previously irradiated field following a 3+ year latency period, whose histology was distinct from the initially irradiated tumour. Clinicopathologic and treatment-related information was extracted from the CanSaRCC database. Overall survival (OS) was defined as the time from RIS diagnosis to death from any cause. Response rate (RR) to neoadjuvant chemotherapy (NACT) was based on physician assessment. Time-to-event analyses were estimated using the Kaplan–Meier method, with Cox regression for multivariate analysis. We considered a two-tailed *p*-value of <0.05 as statistically significant.

**Results:**

One hundred seven tumours met the criteria for RIS and were divided into three subgroups: breast angiosarcoma (BAS, *n* = 54), osteosarcoma (OST, *n* = 16), and other soft-tissue sarcomas (STS, *n* = 37). Patients were mostly female (*n* = 85, 79%), treated initially for breast carcinomas (*n* = 54, 50.5%), and diagnosed with high-grade tumours (*n* = 61/71, 86%). None had evidence of synchronous metastasis. Patients with OST were younger (median age: 48 years, *p* < 0.001), and BAS had the shortest latency interval (8 *versus* 18 years for OST/STS, *p* < 0.001). Most patients underwent surgery, 76% (*n* = 76/100) R0; 24% (*n* = 26) received radiation therapy, mostly (*n* = 15, 57.7%) neoadjuvant. Among those receiving chemotherapy, 30 (75%) underwent NACT; among patients with documented response assessment, the RR was 68% (*n* = 17/25), being even higher in the BAS population (89.5%, *n* = 13/17). Median OS was 53 months (95% CI 34–101), with a 5-year OS of 47.6%; larger tumour size, high histologic grade and older age were independent prognostic factors for worse OS.

**Conclusion:**

Surgery is standard, and NACT might be useful to downsize large lesions, especially in BAS patients. Raising RIS awareness is fundamental to promoting appropriate management and fostering research through multi-institutional collaborations.

## Introduction

Radiation-induced sarcomas (RIS) are highly heterogeneous and account for roughly 3% of all sarcomas.^1^ As per modified Cahan criteria, diagnosis requires a (1) histologically confirmed sarcoma that is distinct from the index tumour (IT), (2) arising in (or nearby) a previously irradiated field (3) after a latency period between radiotherapy and tumour manifestation. The cumulative risk for the development of RIS is estimated at 0.03–0.8% at 10 years. Their rarity has prevented randomized trials to determine an optimal management strategy.^1–4^

To date, *en bloc* resection with negative margins remains the mainstay primary therapy for most soft-tissue sarcomas (STS), including RIS, amenable to upfront surgery. RIS present a challenge to that standard when they have become large due to delayed diagnosis, which can occur due to soft-tissue alterations commonly encountered in irradiated tissues that mask their appearance on physical exam or imaging.^3,5,6^ Consequently, discordant rates of R0 resection have been described in the literature, ranging from 37% to 62%, highlighting the importance of RIS awareness and of management from reference centres specialized in sarcomas.^2,7,8^ The role of neoadjuvant chemotherapy or re-irradiation remains unclear. Unfortunately, RIS overall prognosis remains poor when compared to non-RIS STS, with 5-year survival rates of 30–45%, as opposed to roughly 60% for the latter.^2,4^

Collectively, discordant data, poor prognosis and the absence of randomized clinical studies defining a standard-of-care approach justify the importance of reporting real-world data from high-volume centres to help guide consensus recommendations. We, therefore, sought to characterize the clinico-demographic profile and outcomes of RIS in a cohort of patients available in the Canadian Sarcoma Research and Clinical Collaboration (CanSaRCC) database.

## Methods

### Criteria and sample selection

We defined RIS as arising within a previously irradiated site following a latency period of 3+ years, whose histology was different from that of the index tumour/first malignancy (IT). We retrospectively extracted clinicopathologic data available in the CanSaRCC database for patients diagnosed with RIS between 1996 and 2021, treated in three reference centres in Canada: Princess Margaret Cancer Centre, Mount Sinai Hospital and The Hospital for Sick Children (SickKids).

### Clinicopathologic data

IT-related data comprised age at first RT, tumour lineage (breast carcinoma *versus* sarcoma *versus* other), and RT dose/fractionation; RIS-related data included age at RIS diagnosis, latency period between RT for the IT and RIS diagnosis, RIS histology, grade, maximum diameter, site, surgical resection, margin status, re-irradiation (including dose and fractionation), employment and response to neoadjuvant chemotherapy.

### Outcomes

Clinical outcomes included the physician-assessed response rate (RR), defined as the rate of partial response plus the rate of complete response, freedom from recurrence (FFR), freedom from distant metastasis (FFDM), disease-specific survival (DSS) and overall survival (OS). RR was applied to tumour shrinkage while on neoadjuvant chemotherapy (NACT); FFR was defined as the time from RIS diagnosis to the development of recurrence or progression (for unresectable tumours); FFDM was defined as the time from RIS diagnosis to the development of distant metastasis; DSS was defined as the time from diagnosis to death from RIS. OS was defined as the interval between the date of diagnosis to death from any cause or the date of the last follow-up.

### Statistical analysis

Univariate and multivariate Cox regression models were computed for variables (age, sex, maximum tumour diameter, time from RT to RIS diagnosis, RIS subtype, Federation Nationale des Centres de Lutte Contre le Cancer (FNCLCC) histologic grade, margin status, RT exposure, primary site) correlating with outcomes. OS, DSS, FFDM and FFR were estimated using the Kaplan–Meier method and log-rank test. One-way ANOVA or Kruskal–Wallis with Bonferroni correction for multiple comparisons were used to compare means and medians between groups when deemed appropriate. Chi-square test and Fisher’s exact test were used to compare demographic features. A two-sided *p*-value < 0.05 was considered statistically significant. Statistical analyses were performed using SPSS 28.0 and SAS 9.4.

## Results

### Clinicopathologic and demographic characteristics of the overall population

We identified 107 RIS patients. Eighty-five (79.4%) were females, with a median age at RIS diagnosis of 68.1 years (18.5–89.6). The IT was a sarcoma in 8 (7.5%) cases, and the median latency interval was 10.2 years (3–56.7); the median dose of radiation and number of fractions delivered to the IT were 50 Gy (15–95) and 23.5 (10–50), respectively.

**Figure 1.**
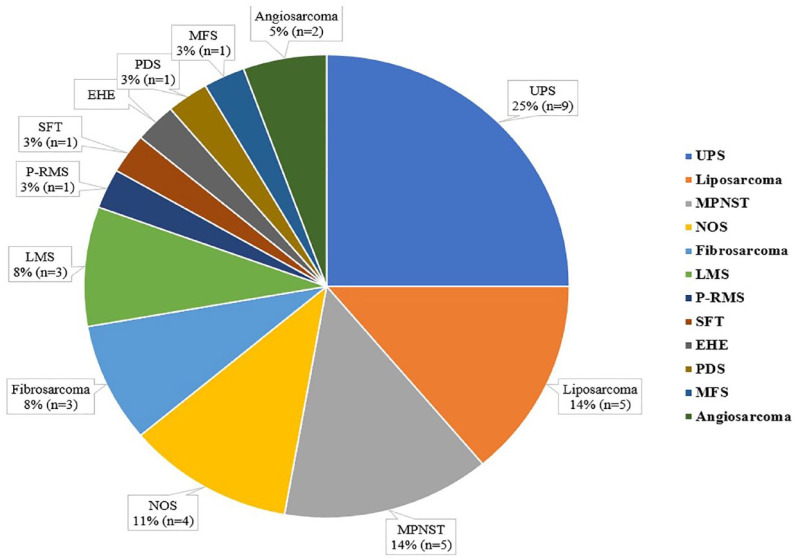
RIS histology – category ‘other STS’ (breast angiosarcoma not included). RIS, radiation-induced sarcomas; STS, soft-tissue sarcomas; UPS, undifferentiated pleomorphic sarcoma; MPNST, malignant peripheral nerve sheath tumor; NOS, not otherwise specified; LMS, leiomyosarcoma; P-RMS, pleomorphic rhabdomyosarcoma, SFT, solitary fibrous tumor; EHE, epithelioid hemangioendothelioma, PDS, pleomorphic dermal sarcoma; MFS, myxofibrosarcoma.

No patient presented with distant metastasis at RIS diagnosis, and the median largest diameter was 5.4 cm (1–23.5). In 65 (60.7%) cases, RIS arose in the breast/chest wall, and most of the patients for whom FNCLCC grading was available were diagnosed with high-grade (61/71 – 86%). The most common RIS was BAS (*n* = 54, 50.4%), followed by osteosarcoma (*n* = 16, 15%), undifferentiated pleomorphic sarcoma – UPS (*n* = 9, 8.4%), liposarcoma (*n* = 5, 4.6%) and malignant peripheral nerve sheath tumour – MPNST (*n* = 5, 4.6%) (Figure 1). More detailed information is available in Table 1.

**Table 1.** Clinico-demographic characteristics by RIS category.

Sarcoma categories	BAS, *n* (%)	OST, *n* (%)	Other STS, *n* (%)	*p*-Value
	*n* = 54 (50.5)	*n* = 16 (15)	*n* = 37 (34.5)	
Age at diagnosis of RIS, median in years (range)	75.5 (47–89.6)	48.4 (18.5–74)	61 (19–85.3)	<0.001
Latency period, median in year (range)	8 (3.8–19.4)	17.4 (4.5–53.6)	18.4 (3–56.7)	<0.001
Sex				<0.001
Male	0	7 (43.8)	15 (40.5)	
Female	54 (100)	9 (56.2)	22 (59.5)	
First malignancy subtype				<0.001
Sarcoma	0	7 (43.8)	1 (2.7)	
Breast carcinoma	54 (100)	0	8 (21.6)	
Other	0	9 (56.2)	28 (75.7)	
RT dose to the first malignancy in Gy, median in years (range)	49.2 (42.4–95)	47.5 (15–78)	47.5 (15–78)	0.09
*n* (%) with available data	21 (38.9)	8 (50)	23 (62.1)	
RT fractionation to the first malignancy	18.5 (16–50)	24 (10–39)	24 (10–39)	0.04
*n* (%) with available data	24 (44.4)	7 (43.7)	21 (56.7)	
RT site (RIS)				<0.001
Breast	54 (100)	0	2 (5.5)	
Head/neck	0	10 (62.5)	3 (8.1)	
Lower/upper extremities/axillae	0	1 (6.3)	10 (27)	
Back/chest wall	0	3 (18.7)	8 (21.6)	
Spine	0	0	0	
Abdomen/pelvis/retroperitoneum	0	2 (12.5)	14 (37.8)	
Largest diameter of RIS, median in cm (range)	4.7 (1.1–19.7)	4.3 (1–8.4)	7.2 (1.8–23.5)	0.025
*n* (%) with available data	52 (96.3)	15 (93.7)	33 (89.2)	
FNCLCC grade				0.84
*n* (%) with available data	31 (57.4)	14 (87.5)	26 (70.3)	
G1	4 (13)	1 (7.1)	5 (19.3)	
G2	10 (32.2)	4 (28.6)	8 (30.7)	
G3	17 (54.8)	9 (64.3)	13 (50)	

BAS, breast angiosarcoma; OST, osteosarcomas.

### Clinicopathologic and demographic characteristics by RIS subgroups

We stratified RIS into three subgroups: breast angiosarcomas (BAS, *n* = 54), osteosarcomas (OST, *n* = 16) and other STS (STS, *n* = 37).

BAS comprised female patients, while the distribution of female *versus* male was closer to proportionate in the OST (56.2%) and STS (59.5%) subgroups. The median age at BAS diagnosis and latency interval were 75.5 (47–89.6) and 8 years (3.8–19.4), respectively, which was the shortest interval among the three subgroups (*p* < 0.001; Table 1). The median largest diameter of BAS was 4.7 cm (1.1–19.7). All BAS patients had breast carcinoma as their IT and had received a median RT dose of 49.2 Gy (42.4–95) in 18.5 fractions (16–50). Among 31 BAS patients with FNCLCC grading available in their charts, 87% were grades 2 or 3 (*n* = 27).

For OST, the median age at diagnosis was 48.4 years (18.5–74), and the median latency interval was 17.4 years (4.5–53.6). The median largest tumour diameter was 4.3 cm (1–8.4). OST patients had received a median RT dose for the IT of 57 Gy (45–66), in a median of 30 fractions (23–33). Roughly 62% developed OST in the head and neck (*n* = 10) (*p* ⩽ 0.001), and most had high-grade tumours (93%, *n* = 13/14).

Lastly, regarding STS, the median age at diagnosis was 61 years (19–85.3), with a median latency period of 18.4 years (3–56.7). The median diameter of the primary tumour was 7.2 cm (1.8–23.5), the largest among all subgroups (*p* = 0.025), and patients had received a median RT dose for the IT of 47.5 Gy (15–78), in 24 fractions (10–39). Abdomen/retroperitoneum followed by extremities/axillae were the most frequent sites of RIS STS, comprising 37.8% (*n* = 14/37) and 27% (*n* = 10/37), respectively (*p* < 0.001). Among 26 patients for whom FNCLCC grading was available, 80.7% (*n* = 21/26) presented with high-grade sarcomas. Unfortunately, germline assessment data was not available. Detailed information about clinicopathologic and demographic characteristics per subgroup is available in Table 1.

### Management of RIS by subgroup

**Figure 2.**
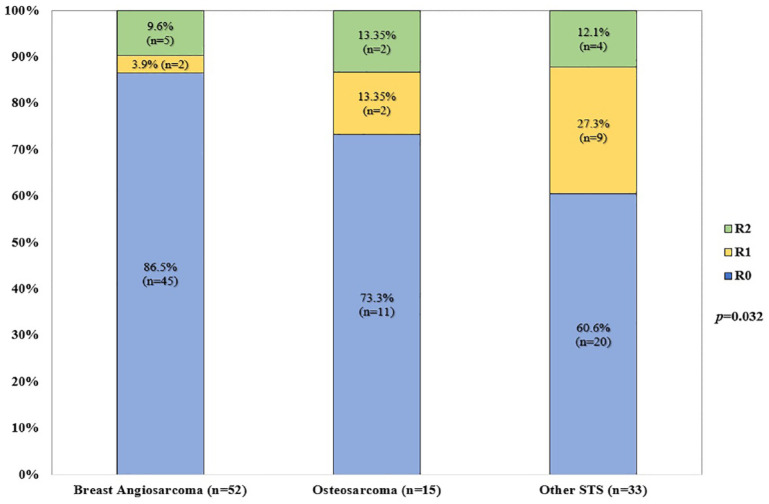
Margin status according to RIS category (*n* = 100). RIS, radiation-induced sarcomas.

Almost all patients underwent surgery for their RIS (*n* = 104, 97.2%), with margins status available for 100 patients. Seventy-six percent (*n* = 76) and 13% (*n* = 13) achieved R0 and R1 resections, respectively; the rates of R0 resection were significantly higher among BAS (*n* = 45/52, 86.5%) and OST (*n* = 11/15, 68.7%) patients, compared with STS (*n* = 20/33, 60.6%) (*p* = 0.032). Figure 2 illustrates the status of surgical margins per subgroup.

Twenty-six patients (24.3%) received RT as part of their RIS management; among those who received it, 57.7% had neoadjuvant RT (*n* = 15/26), and 30.8% had adjuvant treatment. Positive surgical margins occurred in four patients (26.6%) that underwent neoadjuvant RT, with two of them presenting macroscopic involvement.

Regarding the pattern of relapse among the 23 patients who received either adjuvant or neoadjuvant RT, six presented local relapse and four distant relapses; 56.5% remained disease-free during the follow-up period of this study.

Seventy-eight percent (*n* = 18/23) of those who received adjuvant/neoadjuvant RT were in the STS subgroup most had a diagnosis of UPS (*n* = 7/18, 38.9%); these tumours appeared in multiple body sites. The median RT dose was 44 Gy (1.1–60), with a median of 36 fractions (1–50).

Forty patients (37.4%) received chemotherapy, most with neoadjuvant intent (*n* = 30/40, 75%). Among those who received NACT, 19 were BAS, 8 OST and 3 STS (1 MPNST, 1 liposarcoma, 1 UPS); BAS patients received mainly paclitaxel (*n* = 17/19, 89.5%), with only two patients receiving anthracycline-based regimens. Among 17 BAS patients with documented physician’s response assessment to NACT, 13 (76.5%) experienced tumour reduction (5 CR, 8 PR) and 2 experienced disease progression (PD); of the 13 BAS patients experiencing tumour reduction, 12 (92.3%) achieved R0 resection, suggesting a potential role for NACT chemotherapy in this setting. More complete information regarding RIS management and RR are available in Tables 2 and 3, respectively.

**Table 2.** Treatment characteristics and outcomes by RIS category.

Sarcoma categories	Total	BAS, *n* (%)	OST, *n* (%)	Other STS, *n* (%)	*p-*Value
	*n* = 107	*n* = 54 (50.5)	*n* = 16 (15)	*n* = 37 (34.5)	
Surgery					0.64
	Yes	53 (98.1)	15 (93.7)	36 (97.3)	
	No	1 (1.9)	1 (6.3)	1 (2.7)	
Radiotherapy					<0.001
	Neoadjuvant	1 (1.9)	0	14 (37.8)	
	Adjuvant	3 (5.5)	1 (6.3)	4 (10.8)	
	Palliative	0	0	3 (8.2)	
	No RT	50 (92.6)	15 (93.7)	16 (43.2)	
RT dose, median in Gy (range)		47 (6.6–50)	50.96	44 (1.1–60)	N/A
*n* (%) with available data		4 (7.4)	1 (6.25)	18 (85.7)	
RT fractions, median (range)		32.5 (6–40)	N/A	36 (1–50)	N/A
*n* (%) with available data		4 (7.4)	0	18 (85.7)	
Cytotoxic chemotherapy					<0.001
	Yes	23 (42.6)	11 (73.3)	6 (16.2)	
	No	31 (57.4)	5 (26.7)	31 (83.8)	
NACT					0.24
	Yes	19 (82.6)	8 (72.7)	3 (50)	
	No	4 (17.4)	3 (27.3)	3 (50)	
Chemotherapy regimen					<0.001
	VAC	0	0	0	
	Paclitaxel	17 (89.5)	0	0	
	Doxorubicin	1 (5.25)	2 (25)	0	
	Ifosfamide/doxorubicin	0	0	3 (100)	
	Cisplatin/doxorubicin ± MTX	1 (5.25)	6 (75)	0	
Response to NACT					0.84
Missing data: BAS (*n* = 2), OST (*n* = 3)	PD	2 (11.8)	1 (20)	0	
	SD	2 (11.8)	1 (20)	2 (67)	
	PR	8 (47)	3 (60)	1 (33)	
	CR	5 (29.4)	0	0	
Recurrence/progression					0.78
	Yes	30 (55.5)	9 (56.2)	18 (48.65)	
	No	24 (44.5)	7 (43.8)	19 (51.35)	
Outcome					0.46
	NED	19 (35.2)	4 (25)	12 (32.4)	
	Alive with disease	9 (16.7)	2 (12.5)	5 (13.6)	
	Deceased	26 (48.1)	10 (62.5)	20 (54)	

BAS, breast angiosarcoma; CR, complete response; MTX, methotrexate; NACT, neoadjuvant chemotherapy; NED, no evidence of disease; OST, osteosarcoma; PD, progression of disease; PR, partial response; SD, stable disease; STS, soft-tissue sarcoma; VAC: vincristine, doxorubicin, cyclophosphamide.

**Table 3.** Chemotherapy regimens and best response as per physician’s assessment of patients undergoing NACT for RIS.

Patient number	Study ID	RIS category	Chemotherapy regimen	Best response
1	RIS-001	BAS	Weekly paclitaxel	CR
2	RIS-100	BAS	Weekly paclitaxel	CR
3	RIS-034	BAS	Weekly paclitaxel	CR
4	RIS-067	BAS	Weekly paclitaxel	CR*
5	RIS-099	BAS	Weekly paclitaxel	PR
6	RIS-051	BAS	Weekly paclitaxel	PR*
7	RIS-010	BAS	Weekly paclitaxel	PR
8	RIS-085	BAS	Weekly paclitaxel	PR
9	RIS-022	BAS	Weekly paclitaxel	PR
10	RIS-040	BAS	Weekly paclitaxel	PR*
11	RIS-089	BAS	Weekly paclitaxel	PR
12	RIS-102	BAS	Weekly paclitaxel	SD
13	RIS-011	BAS	Weekly paclitaxel	SD
14	RIS-021	BAS	Weekly paclitaxel	PD
15	RIS-053	BAS	Weekly paclitaxel	PD
16	RIS-059	BAS	Weekly paclitaxel	N/A
17	RIS-098	BAS	Weekly paclitaxel	N/A
18	RIS-009	BAS	Doxorubicin single agent	CR
19	RIS-033	BAS	Cisplatin–doxorubicin	PR^†^
20	RIS-107	OST	Cisplatin–doxorubicin	PR
21	RIS-025	OST	Cisplatin–doxorubicin	PR
22	RIS-083	OST	Cisplatin–doxorubicin	PR
23	RIS-077	OST	Cisplatin–doxorubicin	SD
24	RIS-061	OST	Cisplatin–doxorubicin	PD
25	RIS-016	OST	Cisplatin–doxorubicin	N/A
26	RIS-047	OST	Doxorubicin single agent	N/A
27	RIS-006	OST	Doxorubicin single agent	N/A
28	RIS-023	STS	Ifosfamide–doxorubicin	PR
29	RIS-075	STS	Ifosfamide–doxorubicin	SD
30	RIS-069	STS	Ifosfamide–doxorubicin	SD

BAS, breast angiosarcoma; N/A, information not available; NACT, neoadjuvant chemotherapy; OST, osteosarcoma; RIS, radiation-induced sarcomas; STS, other soft-tissue sarcomas.

* Response categorization based on clinical observation (skin examination) only – lesion not well appreciated on conventional imaging.

† Discordant clinico-radiology *versus* pathologic findings – PR on clinical assessment *versus* pathologic complete response.

### Survival outcomes

**Figure 3.**
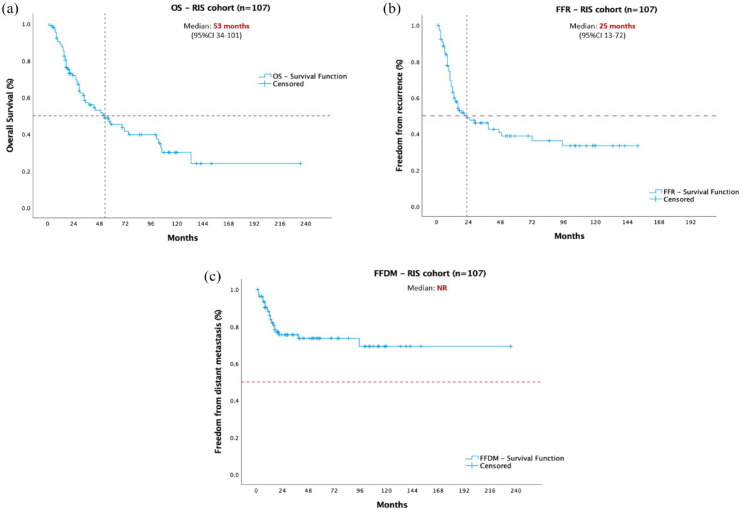
Time-to-event plots illustrating outcomes of the entire RIS cohort. Panel (a) OS in months; (b) FFR in months; (c) FFDM in months. FFR, freedom from recurrence; FFDM, freedom from distant metastasis; OS, overall survival; RIS, radiation-induced sarcomas.

Median OS was 53 months (95% CI 34–101) and the 5-year rate of OS was 47.6% [Figure 3(a)]. On univariate analysis: older age at diagnosis, FNCLCC grade 3, and larger tumours were associated with less favourable outcomes. These findings were corroborated by multivariate analysis (Table 4). Regarding FFR, the median time-to-event was 25 months (95% CI 13–72), with a 5-year FFR of 40% [Figure 3(b)]. None of the clinicopathologic and demographic characteristics were associated with outcomes in the univariate and multivariate analyses. FFDM median survival was not reached (24 events in total) [Figure 3(c)]. On univariate analysis, female gender (HR 3.46, 95% CI 1.53–7.83, *p* = 0.003) and breast as the primary site (HR 2.8, 95% CI 0.9–8.2, *p* = 0.03) were associated with a better prognosis; however, these findings were not confirmed in the multivariate analysis.

**Table 4.** Univariate (UVA) and multivariate (MVA) analyses for overall survival in the entire cohort of RIS patients.

Variables	*n*	UVA *p*-value	MVA-HR (95% CI)	*p*-Value
Age	107	0.03*	2.35 (1.05–5.30)	0.038*
<60	40			
⩾ 60	67			
Sex	107	NS		
Female	85			
Male	22			
Maximum tumour diameter	100	0.04*	1.085 (1.01–1.16)	0.019*
Latency interval	107	NS		
RIS subtype	107	NS		
BAS	54			
OST	16			
STS	37			
Margin status	100	NS		
R0	76			
R1/R2	24			
FNCLCC grade	71			
G1 *versus* G2	10; 22	NS	4.076 (1.06–15.58)	0.04*
G1 *versus* G3	10; 39	0.03*	6.950 (1.82–26.45)	0.0045*

BAS, breast angiosarcoma; NS, non-significant; OST, osteosarcoma; STS, other soft-tissue sarcomas.

* Statistically significant p-value.

We separately assessed the survival outcomes of patients who underwent NACT and presented either partial or complete responses. Among the 30 patients undergoing NACT, 25 had tumour response information available; the median OS for responders *versus* non-responders was 69 (95% CI 1.01–136.1) and 26 months (95% CI 0–55.1, *p* = 0.102), but the small sample size and number of events per subgroup may have affected our ability to detect statistical significance. The same phenomenon was observed for FFR and FFDM, mainly due to a low number of events among patients who underwent NACT, irrespective of their response category. DSS was calculated for the largest and most homogenous subgroup of patients: BAS (BAS-DSS). The median BAS-DSS was 69 months (95% CI 37.3–100.7), with a 5-year BAS-DSS rate of 52.2%.

## Discussion

RIS remains a significant challenge, with retrospective literature suggesting inferior therapeutic outcomes and poor survival when compared to historical data on non-radiation-associated sarcomas.^9–11^ In the absence of clinical trials designed to define optimal RIS management, real-world data from reference sarcoma centres has inordinate value. In this publication, we provided therapeutic and prognostic perspectives of RIS from high-volume Canadian hospitals through the CanSaRCC database.

Our patient cohort consisted predominantly of female patients (71%) whose IT were breast carcinomas, as opposed to sarcomas or other malignancies (58%, *p* < 0.001). Patients with RIS OST were significantly younger (median age: 48.4 years, *p* < 0.001) than the other subgroups, with IT that are more commonly diagnosed in the paediatric and AYA populations (56.2% being either rhabdomyosarcoma, Ewing sarcoma, fibrosarcoma, or retinoblastoma). Similarly, the median age of the BAS population (75.5 years) reflects a latency after the median age of breast cancer patients. There was a significant difference between the median latency period of BAS compared with OST or STS (8 *versus* roughly 18 years for OST/STS, *p* < 0.001).^5,6,9,11–15^ In a publication by D’Angelo *et al*. comprising 79 RIS BAS, the median latency interval was 7 years^14^; Mito *et al*. also reported a significantly shorter latency interval for RIS BAS compared to other-RAS (8 *versus* 15 years).^15^ With regard to body site, the head and neck was significantly more often involved with OST (62.5%, *p* < 0.001), whereas the abdomen and retroperitoneum were more often associated with other STS. Interestingly, patients with other STS had larger tumours at diagnosis (7.2 *versus* 4.0–5.0 cm for OST and BAS, *p* = 0.025) (Figure 2), which might reflect a delayed diagnosis for intra-abdominal sarcomas compared with lesions arising in more superficial areas of the body. In concordance with previous publications, most patients were diagnosed with high-grade RIS, irrespective of their subgroups (86%, *p* = 0.84).

Nearly all patients (97%) underwent surgery as the primary modality, with 76% achieving negative microscopic margins (*n* = 76/100); this data is in line with other publications from high-volume centres.^11,13,15^ The R0 resection rate was higher in the BAS cohort compared with other STS (86.5% *versus* 60.6%, *p* = 0.032). This might reflect differences regarding surgical accessibility of the RIS primary site (breast *versus* abdomen/retroperitoneum) and a larger median tumour diameter at the time of diagnosis. Only 25% of the patients were treated with re-RT, most of them in the other STS subgroup and with neoadjuvant intent (*n* = 14/26, 53.8%, *p* < 0.001).

Most patients received neoadjuvant as opposed to adjuvant chemotherapy and the preferred regimen varied among the three cohorts. RR was 56% (*n* = 17/30) and was higher for BAS patients (76.5%), where one case of pathologic complete response was also observed (RIS-033 – Table 3); these patients were mostly treated with weekly paclitaxel, and we suggest that this regimen could play an important role preoperatively in reducing disease burden and treating micrometastatic disease. Due to a low number of STS and OST patients who underwent NACT, RR estimates are too limited from which to draw meaningful conclusions.

Interestingly, OS estimates for our cohort resemble data published for RIS patients treated with curative intent that homogeneously achieved R0 resections, with a 5-year OS in the range of 40–50% and median OS of 53 months (95% CI 34–101), even though not all patients of our study had negative margins (non-R0 resection: 24%)^2^; we identified older age, larger tumour size and FNCLCC grades two-thirds as independent prognostic factors for inferior OS, validating previous studies. However, as opposed to other publications, margin status and disease were not significant correlates in our analysis, and we believe that the relatively small sample size of OST and STS subgroups may have impacted our ability to detect existing differences.^3,11–13,15,16^ BAS patients accounted for 50% of the study population and 86% of them had negative margins after curative surgery.

We acknowledge that mixing different sarcoma subgroups is one potential limitation of our publication, though common in RIS literature due to the rarity of these diseases. In addition, the absence of matched internal controls comprised of non-RIS, at least for the most frequent histologies, limits comparisons between RIS subtypes and their non-RIS counterparts. These analyses are important since they provide prognostic information for specific RIS subtypes that can potentially direct RIS-specific therapy. Gladdy *et al*. reported inferior DSS for radiation-induced UPS when compared to a matched cohort of sporadic tumours with the same histopathologic diagnosis (5-year DSS 44% *versus* 66%, respectively)^13^; more recently, Bartlett *et al*., described worse 5-year disease-specific death (DSD) rates for radiation-induced MPNST patients in comparison with the ones diagnosed with sporadic/non-NF-1-associated MPNST (38% *versus* 75%, respectively), but no statistically significant DSD rate discrepancies were observed for other sarcomas such as UPS, myxofibrosarcoma and leiomyosarcoma, in the same study.^11^ Understanding which RIS histologies fare worse when compared with sporadic sarcomas may be of value in the discussion of when patients should ideally be less aggressively treated.

Secondly, the small number of OST and STS patients limits our ability to detect important prognostic information about these subgroups. Moreover, the lack of germline mutational status registered in the database did not allow for further investigation regarding the relationships between germline pathogenic variants and the incidence/outcomes of RIS patients, especially for those living with more frequent cancer predisposition syndromes such as the Li–Fraumeni syndrome and Familial Adenomatous Polyposis.

Despite these limitations, our study includes one of the largest series of RIS, presenting valuable prognostic information and treatment inputs from two reference sarcoma institutions in Canada. The high rate of R0 surgery reflects expertise in sarcoma management from these hospitals, underscoring the importance of patient referral to high-volume cancer centres to treat these rare malignancies in a multidisciplinary way.^17,18^ Further, by summarizing the available data regarding NACT outcomes and a compelling RR for BAS, the present publication suggests that these patients should be considered for neoadjuvant weekly paclitaxel for borderline resectable cases during multidisciplinary boards. Though our study failed to demonstrate a statistically significant association between negative margins and superior survival outcomes (maybe due to the high percentage of R0 resections), it is reasonable to consider this possibility since other large series have concordantly suggested that. To the best of our knowledge, our cohort provides response assessment for the largest number of RIS patients exposed to NACT ever reported.

Looking ahead, attempts to characterize molecular alterations that are linked with RIS development may contribute to uncovering prognostic and predictive biomarkers, enabling therapeutic innovation for this group of patients. *MYC* amplifications and positive immunostaining have been observed in all 25 cases of RIS BAS reported by Mentzel and colleagues,^19^ contrasting with non-radiation-induced angiosarcomas.^20,21^ Furthermore, Thibodeau *et al*. described recurrent missense variants in *EGFR*, *BRAF* and homologous recombination repair genes, such as *BRCA1*, in a cohort of 13 RIS BAS patients^22^; besides, a predominance of C→T substitutions was observed, a feature that has been previously linked with response to anti-PD-1 antibodies in cutaneous melanomas.^23^ Malone *et al*. reported *HRAS* (*n* = 2) and *FGFR4* (*n* = 2) pathogenic or likely pathogenic variants among samples of 12 patients with diverse RIS histologies that underwent whole exome sequencing; a 45% prevalence of PD-L1 positivity (set as immunostaining in ⩾1% of the tumour cells) among samples of 20 individuals, along with the presence of CD4+ and CD8+ tumour-infiltrating lymphocytes (threshold ⩾11 cells/10HPF) in 15% and 20%, respectively, also suggests vulnerability to anti-PD-1/PD-L1 immune-checkpoint inhibition (ICI) in a subset of RIS patients.^24^ Clinical activity of ICI in RIS has been illustrated in the publication of Florou *et al*., where the authors reported a case of metastatic RIS BAS that achieved sustained PR on pembrolizumab following disease progression upon anthracycline-based chemotherapy, gemcitabine/docetaxel and pazopanib.^25^ It is reasonable to believe that comparative analyses of genomic and tumour immune infiltrate profiles may be helpful to elucidate not only to what extent certain RIS histologies differ from their non-radiation-induced counterparts but also if similar immunotherapy responses can be expected, especially in specific sarcoma subtypes for which preliminary encouraging activity has been suggested (e.g. UPS, angiosarcoma, liposarcoma).^26–32^

Accelerating advances in the understanding, prognostication and therapeutic strategies of RIS will only be possible by raising RIS awareness among patients and the overall medical community, which entails having a lower threshold for suspicion in cancer survivors treated with RT that present with otherwise unclear clinical findings in radiated sites. A direct communication approach must exist between non-Oncology physicians following these patients in the long-term and reference centres in the management of sarcomas to enable appropriate investigation, multidisciplinary management, research collaborations and access to clinical trials whenever available.

## Conclusions

RIS are rare and heterogenous entities, for which surgery remains the modality of choice when pursuing treatment with curative intent. NACT should be considered for larger lesions, especially in cases of BAS and management should ideally be discussed in multidisciplinary boards. Large tumours, older age and high histologic grade are independent prognostic factors for inferior OS. Raising RIS awareness is fundamental to promoting appropriate patient management and fostering research through multi-institutional collaborations.

## Declarations

**Ethics approval and consent to participate:** Patients provided written consent allowing their personal information to be stored and extracted from the CanSaRCC database for studying purposes (master CanSaRCC protocol REB 10-0804). The present project was also approved by our local institutional review board (REB 20-5520).

**Competing interests:** M.F.R. – honoraria: Bristol Myers Squibb. G.W. – honoraria: Pfizer, Gilead Sciences; consulting/advisory role: Novartis; travel grants: Abbvie. A.A.R. – consulting/advisory role: Adaptimmune, Bayer, GlaxoSmithKline, Inhibrx, Medison; expert testimony: Medison; research funding: 23andMe, Abbvie, Adaptimmune, Amgen, Blueprint Medicines, Boehringer Ingelheim, Bristol Myers Squibb, Daiichi-Sankyo, Deciphera, Frontier Therapeutics, GlaxoSmithKline, Iterion Therapeutics, Karyopharm Therapeutics, MedImmune, Merck, Neoleukin Therapeutics, Pfizer, Rain Therapeutics, Roche/Genentech, Symphogen. H.P.S., Z.B., I.H., J.W., P.F., K.T., S.B., R.G., C.S., P.C., C.C., P.W., A.A.G., D.S. – no competing interests to disclose.

**Availability of data and materials:** Access to specific data and analyses can be requested through cansarcc@uhn.ca.
